# Galangin Protects against Symptoms of Dextran Sodium Sulfate-Induced Acute Colitis by Activating Autophagy and Modulating the Gut Microbiota

**DOI:** 10.3390/nu12020347

**Published:** 2020-01-29

**Authors:** Hongzhuan Xuan, Aiqun Ou, Shengyu Hao, Jiajun Shi, Xiaolu Jin

**Affiliations:** 1 School of Life Science, Liaocheng University, Liaocheng 252059, China; hongzhuanxuan@163.com; 2 State Key Laboratory of Animal Nutrition, College of Animal Science and Technology, China Agricultural University, 2 Yuanmingyuan West Road, Beijing 100193, China; 3 College of Bee Science, Fujian Agriculture and Forestry University, Fuzhou 350002, China; ouaiqun2018@126.com; 4 School of Physical Science and Information Technology, Liaocheng University, Liaocheng 252059, China; haoshengyu@lcu.edu.cn; 5 Department of Experimental Animals, Zhejiang Academy of Traditional Chinese Medicine, Hangzhou 310007, China; xsrjsjj1992@126.com

**Keywords:** galangin, ulcerative colitis, autophagy, gut microbiota, short-chain fatty acid

## Abstract

Galangin is a natural flavonoid that has been reported to provide substantial health benefits. Nevertheless, little is known about the potential effects of galangin against inflammatory bowel diseases. Here, an in vivo study was performed to investigate the preventive effects of galangin against dextran sulphate sodium (DSS)-induced acute murine colitis, which mimics the symptoms of human ulcerative colitis (UC). Pre-treatment with galangin (15 mg/kg, *p.o.*) resulted in a significant decreased in the macroscopic signs of DSS-induced colitic symptoms, including a decreased disease activity index, prevention of the colon length shortening, and alleviation of the pathological changes occurring in the colon. Colonic pro-inflammatory mediators, including tumor necrosis factor-alpha, interleukin (IL)-1 beta, and IL-6, as well as myeloperoxidase activities were decreased following galangin pre-treatment when compared with the DSS control group. Moreover, galangin pre-treatment significantly increased the expressions of autophagy-related proteins and promoted the formation of autophagosome in the colon. Galangin pre-treatment increased the diversity of the gut microbiota, and this was accompanied by increased levels of short-chain fatty acids. These observed changes could involve the modulating effects conferred by galangin in relation to some specific bacteria populations, including the recovery of *Lactobacillus* spp., and increased *Butyricimonas* spp. Overall, these results support the use of galangin in the prevention of UC.

## 1. Introduction

Inflammatory bowel diseases (IBD) consist of two partially overlapping clinical entities, Crohn’s disease (CD) and ulcerative colitis (UC), which have demonstrated a rapidly increasing incidence worldwide [1]. CD typically presents as scattered transmural inflammation of the patients’ gastrointestinal (GI) tract, whereas mucosal inflammation in patients with UC is limited to the colon [2]. Although the pathogenesis of IBD remains unknown, several therapeutic approaches, which mainly focus on immunosuppression and anti-inflammation, have been developed. However, patients with IBD receiving such treatments often do not achieve full remission and have a high possibility of recurrence. In total, one-fifth of patients with CD and one-tenth of patients with UC ultimately require colectomy surgery, which increases the burden on the healthcare system and economy [3].

The microbes within the gut have versatile roles in maintaining the host’s health, and dysbiosis of the gut microbiota (e.g., decreased bacterial diversity and richness and/or an imbalanced bacteria composition) is also associated with the occurrence [4]. Such microbes regulate immune homeostasis in the intestinal epithelium and also produce secondary metabolites, such as short-chain fatty acids (SCFAs) [5]. Nevertheless, under pathological conditions, indigenous bacteria can accelerate disease development and are considered external pathogens. Dysbiosis of the gut microbiota is characterized by a significant reduction of obligate anaerobes (phyla Bacteroidetes and Firmicutes) and increase in facultative anaerobes (phyla Actinobacteria and Proteobacteria, such as *Escherichia coli*), which results in increased inflammation [6]. As such, it is widely recognized that the reshaping of a disrupted gut microbiota might serve as an effective strategy for the prevention and/or treatment of IBD [4]. Since diet is the key environmental factor that serves to modulate the composition of the gut microbe and contributes to the incidence of IBD, increasing interest has been focused on using therapeutic constituents from dietary sources (including fiber, fruits, and vegetables) [7].

Flavonoids are a major group of secondary plant metabolites with variable phenolic structures. Flavonoids are also the most widely distributed phenolic compounds among all the parts of the plant, and these have been shown to confer a myriad of biological and pharmacological effects [8]. Flavonoids are also regarded as the major active constituents of bee propolis, a natural dietary supplement produced by honeybees from the resin of plants which has been widely used to prevent inflammatory diseases [9]. Recent studies from our lab have also suggested that propolis flavonoids have considerable potential as complementary and alternative reagents relative to classical IBD therapeutics [10,11]. Nevertheless, propolis contains numerous flavonoids, and there is scant information regarding the active constituents in relation to its gastrointestinal effects. Galangin (3,5,7-trihydroxyflavone) is one flavonol identified as a major active phytochemical in propolis and *Alpinia officinarum Hance* (Galangal) [12]. Recent studies have shown that galangin confers some effects towards suppressing the pathogenesis of UC induced by dextran sulphate sodium (DSS) via the inhibition of inflammation [13]. Even though most flavonoids present suitable anti-inflammatory effects, the specific mechanisms of action underlying the amelioration of UC by galangin still require further analysis.

Galangin functions as an important natural autophagy inducer and has attracted growing attention in recent years. Autophagy is a regulated self-digestion process with great significance in maintaining intestinal homeostasis. Emerging data have suggested that some IBD-associated genes, such as autophagy-related (ATG)16L1 and IRGM have functional importance in relation to antibacterial autophagy in patients with IBD susceptibility [14]. To date, the roles of galangin at the autophagic level during IBD are still unknown. In this study, the protective mechanisms conferred by galangin were assessed with an emphasis on the induction of autophagy and regulation of the gut microbiota using a DSS-induced UC mouse model.

## 2. Materials and Methods

### 2.1. Chemicals

Galangin (purity: ≥99%) was obtained from Biopurify Phytochemicals Ltd. (Chengdu, China). Dextran sulphate sodium (molecular weight: 36,000–50,000) was purchased from MP Biomedicals (Irvine, CA, USA). Primary antibodies against phosphor-AMPK-alpha (p-AMPKα, Thr172), LC3A/B, and β-actin were purchased from Cell Signaling Technology (Danvers, MA, USA). Primary antibodies against ATG5, ATG7, and ATG12 were purchased from Proteintech Group, Inc. (Chicago, IL, USA). Horseradish peroxidase (HRP)-conjugated secondary antibody (anti-rabbit IgG) were purchased from HuaAn Bio-Technology Co., Ltd. (Hangzhou, China). All other chemicals were obtained from Sigma-Aldrich (St Louis, MO, USA).

### 2.2. Animal Experiments

Male ICR mice (7 weeks old, 22-24 g) were purchased from Shanghai Laboratory Animal Research Center (Shanghai, China). This study was conducted in the Animal Experimental Center of the Zhejiang Institute of Traditional Chinese Medicine in Hangzhou, China, following standard experimental protocols in the SPF environment. The experiment was carried out in accordance with the code for the care and use of animals for scientific purposes (ethic approval code: 171003). Mice were acclimatized to standard laboratory conditions at 23 °C, 12 h/12 h light/dark, and 50% humidity for 4 days prior to the experiment. An AIN-93-based standard lab chow (Xietong Biotechnology, Nanjing, China) was applied to the animals, and all of the mice had free access to feed and water throughout the experimental period. The mice were randomly divided into four treatment groups of equal size (*n* = 6/group). The treatment groups were: (1) normal control, which received tap water and were not treated with DSS; (2) the DSS colitis control, which received 3% DSS in tap water for 1 week; (3) positive control group, in which mice received 5-aminosalicylic acid (50 mg/kg b.w., *p.o.*); and (4) galangin treatment group, in which mice received galangin (15 mg/kg b.w., *p.o.*). Galangin and 5-aminosalicylic acid were dissolved in 0.5% gum tragacanth (solubilizing agent), and administered to the mice 1 week prior to the DSS challenge, up to the end of this study. The normal control and DSS colitis control mice also received the same volume of gum tragacanth. The effect of a treatment on an animal’s health was blindly analyzed and the disease activity index (DAI) was chosen for evaluating the colitic severity, which included the loss of body weight, stool consistency, rectal bleeding, and the overall condition of the animals [11].

### 2.3. Histologic Analysis

Distal colonic tissues were collected from the mouse and the digesta were removed. These tissues were fixed in 4% paraformaldehyde for hematoxylin and eosin (H&E) staining following standard protocols. Colonic histological tissues were observed under a light microscope with an attached image capture system (Nikon Eclipse Ci, Japan).

Histological damage to the colon were calculated on the basis of colonic inflammation, the severity of colonic epithelial cell infiltration, crypt destruction, and the extent of cell infiltration, with each of the subscore ranging from 0 to 2 (no changes to maximum tissue damage).

### 2.4. Inflammatory Cytokines Assessments

Colonic proteins were extracted using a RIPA cell lysis buffer, and pro-inflammatory cytokines, tumor necrosis factor (TNF)-α, interleukin (IL)-1β, and IL-6, were measured using commercial enzyme-linked immunosorbent assay kits following standard protocols (Cusabio Biotech, Wuhan, China). Colonic myeloperoxidase (MPO) peroxidase activity was measured on the basis of a previously described colorimetric method, with some modifications [15]. In brief, colonic tissues were extracted using PBS (0.1M, pH = 6.5) with 0.5% hexadecyltrimethylammonium bromide. Then, the reaction solution (0.167 mg/mL of o-dianisidine hydrochloride, 0.0005% hydrogen peroxide) was mixed with the tissue homogenate (0.1 mL with 2.9 mL reaction solution) and then reacted for 5 min. The optical values were subsequently recorded at 460 nm (Molecular Devices, Spectramax M5).

### 2.5. Western Blot Analysis

Colonic proteins were obtained using a commercial protein extraction kit (KeyGen Biotech, Co, Ltd., Nanjing, China). Protein concentration was determined using a BCA protein assay kit (Beyotime Biotechnology, Haimeng, China). Subsequently, equal amounts of proteins (40 μg) were mixed with a quarter-volume of the Laemmli’s sample buffer and boiled at 95 °C for 5 min. These samples were then separated via 12–15% SDS-PAGE. The gels were then transferred to polyvinylidene fluoride (PVDF) membranes (Millipore, USA), and 5% skim milk was used to block the nonspecific binding sites for 30 min at room temperature. Primary antibodies (as indicated) were incubated with the membranes at room temperature for 1 h, and horseradish peroxidase (HRP)-conjugated secondary antibodies were then applied for another 1 h incubation. The blots were visualized using ECL Western blotting detection reagents (Abcam, Cambridge, MA, USA).

### 2.6. Transmission Electron Microscopy

For the transmission electron microscopy (TEM) analysis, distal colon samples were fixed using glutaraldehyde (2.5%) and 1% osmium tetroxide (1%) in 0.01 M phosphate. The fixed tissues were then embedded in Epon 812 resin overnight and dried with an acetone gradient following the standard procedures provided by the manufacturer (SPI-EM, Division of Structure Probe, Westchester, NY, USA) [16]. The ultrathin sections were then dried using uranium acid and lead citrate, and subsequently observed under a transmission electron microscope (Hitachi, Tokyo, Japan).

### 2.7. Gut Microbiota Analysis

Cecal digesta were collected, and total DNA was extracted using a QIAamp DNA stool MiniKit (Qiagen, Hilden, Germany). PCR reactions targeting the V3–V4 regions of the 16S rDNA genes (primers: 319F, 5′-ACTCCTACGGGAGGCAGCAG-3′; 806R, 5′-GGACTACHVGGGTWTCTAAT-3′) were performed using the Premix Ex Taq Hot Start Version (Takara, Dalian, China) on a gradient PCR instrument. The amplicons were then purified using an AxyPrep DNA gel extraction kit (Axygen Bioscience, Union City, USA). The sequencing reactions were subsequently assessed using an Illumina Miseq PE250 platform (Shanghai Majorbio Bio-pharm Biotechnology Co., China). Raw reads were merged using FLASH (v1.2.11) and then analyzed using QIIME (v1.9.0). The sequences were binned into operational taxonomic units (OTUs) on the basis of 97% identity. Taxonomy analyses were performed using the Ramer–Douglas–Peucker (RDP) algorithm of the Greengenes database. Alpha diversity indices, such as Chao1, Shannon, and Simpson diversity indices, were chosen to estimate the species richness using the Mothur program. Principal coordinate analysis (PCoA) was used to represent the relationships between the samples on the basis of calculations of UniFrac metrics. Heat map and the hierarchical clustering tree at the genus level was calculated based on a UniFrac metrics dissimilarity matrix using Vegan 2.0 packages in R (version 3.1.2) software.

### 2.8. Short-Chain Fatty Acid Analysis

Weighed cecal digesta (~100mg) collected from the mice were diluted at 1:3 (w/w) with 1.68 mmol heptanoic acid/L, which served as an internal standard. Cecal acetate, butyrate, propionate, and minor SCFAs were measured using a gas chromatography system on the basis of a previously published method [16].

### 2.9. Statistics Analysis

Data are presented as arithmetic mean ± standard deviations (SD) for each treatment group. Depending on the number of variables, normally distributed datasets were either compared by one-way ANOVA or repeated-measures ANOVA followed by Tukey’s post-hoc tests. A *p*-value less than 0.05 was considered statistically significant. All statistical tests were performed using SPSS version 17.0 (SPSS, Inc., Chicago, IL).

## 3. Results

### 3.1. Galangin Ameliorated AC Symptoms Induced by DSS in Mice

**Figure 1:**
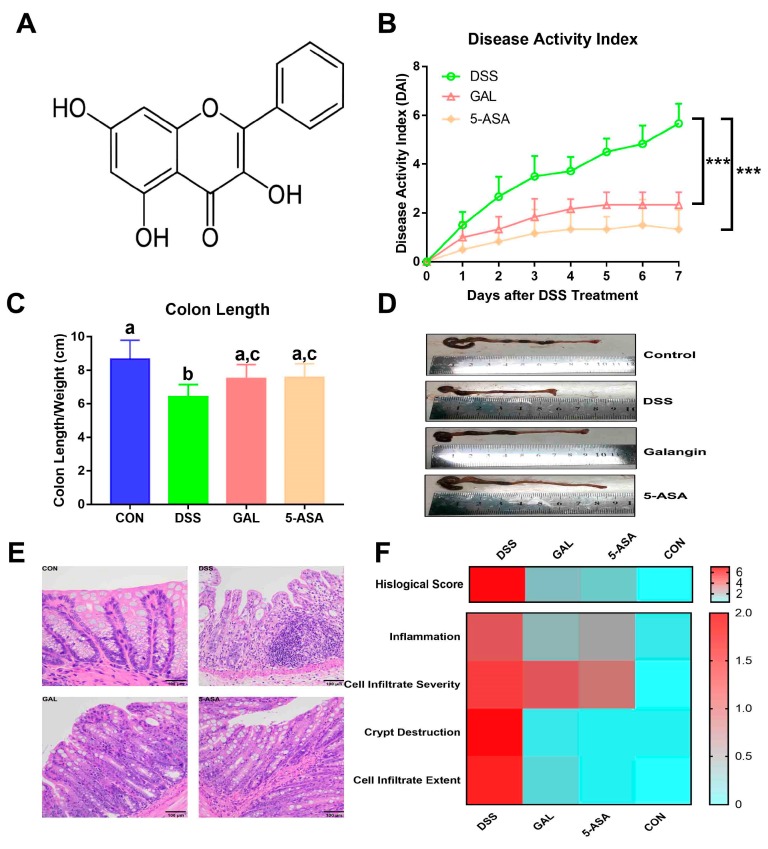
Galangin (GAL) ameliorated acute colitis symptoms induced by dextran sulphate sodium (DSS) in mice. Galangin (15mg/kg, *p.o*.) were pre-administrated to the mice for 7 days and until the end of the study. DSS (M.W. 36–50 kDa, 3% in tap water) was applied to the animals for 1 week. (**A**) Chemical structure of galangin. (**B**) Effects of galangin on the disease activity index, which was described in the Material and Methods. Data are expressed as the mean ± SD (*n* = 6). Statistic difference between groups was calculated on the basis of repeated-measurement ANOVA. *** *p* < 0.001. (**C**) Effects of galangin on colon length changes. Groups with different letters differ by a statistically significant margin (*p* < 0.05). (**D**) Representative pictures showed macroscopic features of colon tissues. (**E**) Representative pictures showed histopathological features of colon tissues. (**F**) Heatmap representation of histological scores, which includes colonic inflammation, colonic epithelial cell infiltration severity, crypt destruction, and cell infiltration extent. Each row shows one individual index and each column an experimental group. High histological scores are shown in red and low histological scores in blue. CON, control group; DSS, DSS colitis group; GAL, galangin group; 5-ASA, 5-aminosalicylic acid group.

As shown in Figure 1B, a dramatic increase in the DAI following DSS administration (3% in tap water) over 1 week was observed, including decreased weight, stool consistency, and overall condition of the animals, suggesting that the DSS treatment resulted in symptoms similar to that of clinical UC. In comparison with the DSS control group, it can be been seen that the DAI values were significantly inhibited following the galangin and the 5-aminosalicylic acid (5-ASA, reference drug) treatments (both *p* < 0.001), which corroborate their protective effects against DSS-induced colitis. The colon lengths were substantially shortened in the DSS groups, and this was rescued following galangin treatment (Figure 1C,D). Correspondingly, distal colonic tissues from the DSS-induced colitis group presented with massive crypt disruptions, ulcerations, and severe inflammation, based on the results of H&E staining (Figure 1E). These pathological changes were alleviated by the administration of galangin or 5-ASA, which further confirmed the decreasing H&E scores from the semi-quantitative scores of the histological parameters (Figure 1F).

### 3.2. Effects of Galangin on Inflammatory Mediators in DSS-Induced Colitic Mice

**Figure 2:**
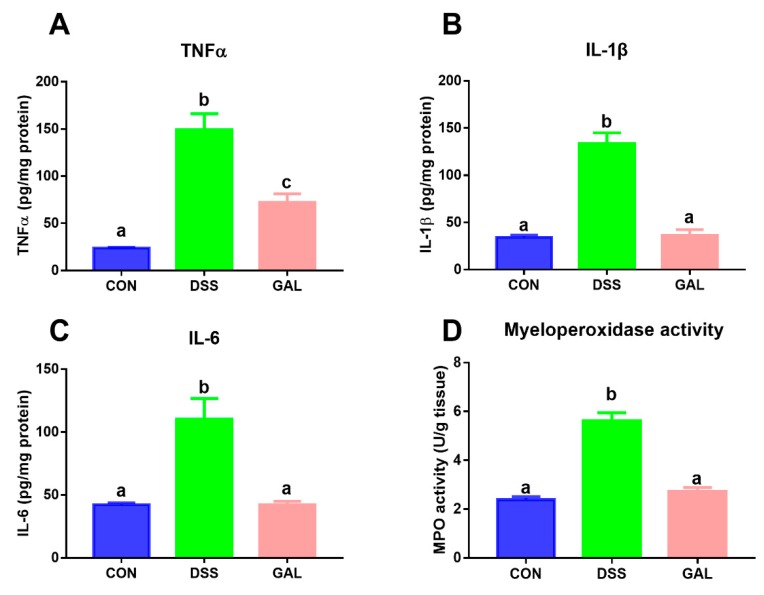
Effects of galangin administration on inflammatory mediators in the colons from DSS-induced colitic mice. (**A**) Tumor necrosis factor (TNF)-α, (**B**) interleukin (IL)-1β, (**C**) IL-6, and (**D**) myeloperoxidase (MPO) levels in the colonic tissues. The values are expressed as mean ± SD (*n* = 6). Means sharing the same letter are not significantly different from each other (*p* < 0.05).

In comparison with the control group, the levels of TNF-α, IL-1β, and IL-6 in the DSS groups were clearly increased. Importantly, treatment with galangin resulted in a decrease in these pro-inflammatory cytokines when compared with the DSS colitis control (Figure 2A–C). Galangin treatment also significantly reversed the increase in the colonic MPO activity when compared to the DSS-treated mice (Figure 2D).

### 3.3. Galangin Administration Activated Cellular Autophagy in DSS-Induced Colitic Mice

**Figure 3:**
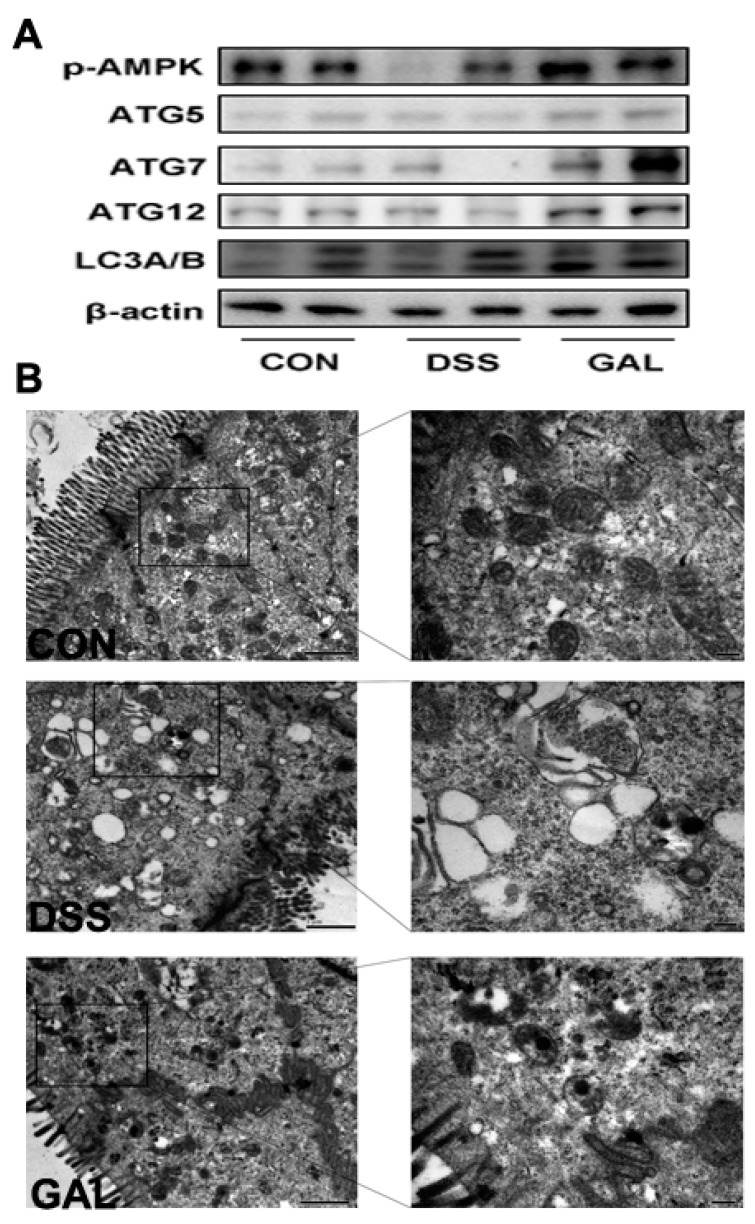
Galangin administration activated cellular autophagy in colon from DSS-induced colitic mice. (**A**) Mice distal colons were collected from the colitic mice for Western blotting and quantitative analysis of macroautophagy proteins. (**B**) Transmission electronic microscopy analysis on colon samples from DSS-induced colitic mice. Representative photos were taken using TEM of the mice colon from control, DSS, and galangin groups.

The expressions of autophagy-associated proteins in the colons of DSS-induced colitic mice were assessed. As shown in Figure 3A, it was observed that the autophagy marker proteins, including ATG5, ATG7, ATG12, and LC3B, were downregulated in the colitic mice, compared with the control group. Galangin administration promoted colonic cell autophagy by increasing the aforementioned autophagy-associated proteins. In addition, phosphorylated AMPK expression was decreased in the DSS-treated mice, and this response to DSS was largely reversed following galangin administration. According to TEM analyses, significant epithelial barrier losses were observed in the DSS-treated mice, whereas galangin was shown to significantly induce autophagosome formations in the intestinal epithelial cells.

### 3.4. Galangin Administration Modulated the Gut Microbiota in Colitic Mice

**Figure 4:**
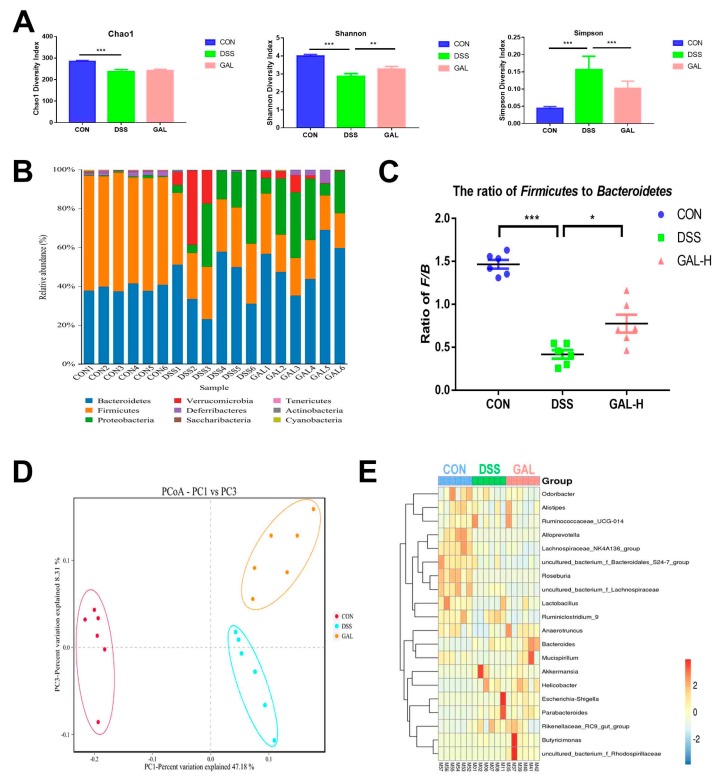
Galangin administration modulated gut microbiota in colitic mice. Mice cecal digesta were collected from the mice for 16S rRNA gene sequencing analysis. (**A**) Gut microbial α-diversity was calculated on the basis of Chao1, Simpson, and Shannon indices. (**B**) Gut microbiota composition at the phylum level. (**C**) The ratio of Firmicutes to Bacteroidetes in the gut microbiota. (**D**) Gut microbial β-diversity was calculated on the basis of weighted UniFrac metrics and presented as the principal coordinate analysis (PCoA) plot. (**E**) Heat map of the 20 most differentially abundant taxons among the control, DSS, and galangin groups at the genus level (* *p* < 0.05, ** *p* < 0.01, and *** *p* < 0.001).

To determine if galangin administration was linked to changes in the gut microbial communities, a high-throughput Illumina MiSeq sequencing approach was employed, which covered the V3–V4 regions of the 16S rRNA gene of the bacteria extracted from the cecal digesta of the colitic mice. As shown in Figure 4A, compared with the normal control, mice from the DSS colitic group showed a remarkable decrease in the community diversity (significant decreases in the Shannon indices and increases in the Simpson indices-both were *p* < 0.001) and an overall loss of community (decreased Chao1 indices, *p* < 0.001). Galangin treatment was found to effectively reverse the decrease in the bacterial community diversity induced by DSS (*p* < 0.01 of Shannon indices, and *p* < 0.001 of Simpson indices); however galangin treatment was not able to increase the community richness. As shown in Figure 4B, the predominant bacterial phylum in the normal control was Firmicutes (57.4%), followed by Bacteroidetes (39.2%). After DSS treatment, Bacteroidetes were increased to 41.2%, and Firmicutes were decreased to 29.3%, representing a significant increase in Proteobacteria. Correspondingly, compared with the normal mice, a decreased ratio of Bacteroidetes to Firmicutes was observed in the DSS group. Moreover, this ratio was increased following the administration of galangin (Figure 4C). The PCoA method was then used to further depict the distinct microbial compositions among the control, DSS, and galangin groups (Figure 4D). A heat map was generated to show the most predominant 20 bacteria genera. It was observed that the control mice had a wide range of bacterial genera, including *Lactobacillus* spp., *Alloprevotella* spp., *Odoribacter* spp., and *Roseburia* spp., among others. However, DSS induction decreased the relative abundance of some probiotic bacteria, including *Lactobacillus* spp., and increased the abundance of the pathogenic bacteria, such as *Escherichia shigella* spp. In addition, treatment with galangin was able to recover the loss of *Lactobacillus* spp., and induce some specific bacterial groups, including *Butyricimonas* spp. and *Mucispirillum* spp. (Figure 4E).

### 3.5. Effects of Galangin Administration on the SCFA Composition in Colitic Mice

**Figure 5:**
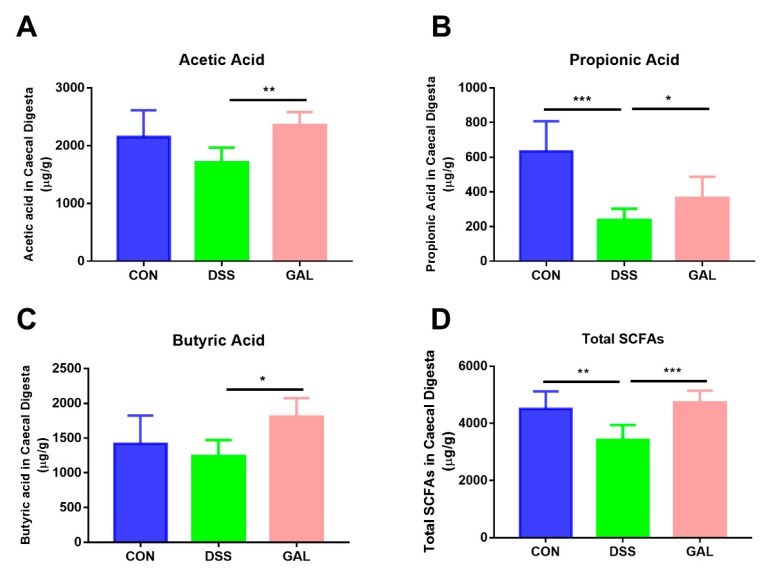
Galangin administration increased gut SFCAs in DSS-induced colitic mice. Feces were collected from mice and SCFAs were analyzed using GC. Results of acetic acid (**A**), propionic acid (**B**), butyric acid (**C**), and total SCFAs (**D**) are shown as µg/g of feces f.w. (fresh weight). The stars indicate that values were significantly different (* *p* < 0.05, ** *p* < 0.01, and *** *p* < 0.001) compared with DSS control.

As shown in Figure 5, the concentrations of fecal SCFAs were determined using GC analysis. A significant decrease in the total SCFAs in the DSS-treated mice when compared with the normal control (*p* < 0.01) was observed. Three major SCFAs, including acetic, propionic, and butyric acid (~90% of total), also presented with a similar decreasing trend. Following galangin treatment, it was apparent that the decrease in the total SCFAs and the three main SCFAs was reversed to some extent compared to the DSS-induced colitic mice, suggesting that galangin intake could help modulate bacterial metabolites in the gut.

## 4. Discussion

Although galangin has been shown to have numerous biological activities, which include key active constituents of many herbs and natural products (including bee propolis [17], *Alpinia officinarum Hance* [18], etc.), studies assessing the gastrointestinal protective effects of galangin are limited. In the present study, we investigated the potential impact of galangin administration against intestinal inflammation using a mouse UC-like model induced by DSS. Galangin administration to these mice was able to prevent the damage induced by intestinal inflammation, as well as limit the clinical colitic symptoms. Moreover, these effects were mediated via the activation of autophagy-related cell signaling pathways. We further confirmed the modulating effects of galangin on the gut microbiota and SCFA production in relation to the attenuation of intestinal inflammation.

The incidence of UC has grown rapidly over recent years. UC can severely decrease a patient’s quality of life and is also a factor influencing the development of colonic cancer [19]. Even though a number of anti-inflammatory/immunosuppressive drugs (e.g., prednisone, azathioprine, and methotrexate) have been developed, patients sometimes do not achieve full remission [20]. In addition, potential side effects, such as steroid dependence, have also been reported to be associated with these therapies [21]. As such, new therapeutic approaches are warranted for the treatment and/or prevention of UC. The UC model used in the current study was induced in mice using DSS over 7 days, which represents one of the best-characterized animal models for IBD for use in pre-clinical studies [22]. Similar to a previously study, we noticed that the DSS treatment caused mucosal barrier losses as well as inflammation in the intestinal epithelial cells, resulting in diarrhea and shortened colons in the mice [23]. Flavonoids, including galangin have good anti-inflammatory activity, and some specific flavonoids, such as pinocembrin [16], naringenin [24], and phloretin [25] have been demonstrated to have good anti-colitis effects. However, other flavonoids, such as luteolin, can aggravate DSS-induced experimental colitis, suggesting that the mechanisms of action associated with flavonoids are quite complex [26]. As expected, it was noticed that the administration of galangin to these mice had beneficial effects towards alleviating the symptoms associated with acute colitis, including a decreasing DAI, increasing colon length, and protection against histopathological changes in the colonic cells. These macroscopic indices indicated that galangin could effectively suppress the clinical features of colitis induced by DSS in mice.

It has been widely accepted that inflammatory cytokines play key roles in the pathogenesis of IBD [27]. The severity, perpetuation, and complications associated with IBD are also directly correlated with the releases of inflammatory cytokines, which originated from the inflammatory cells (epithelial cells, macrophages, and neutrophils) in the colon [28]. Therefore, controlling the productions of inflammatory cytokines has some merits in regard to evaluating the effectiveness of UC or CD therapies. In clinical practice, specific antibodies against some pro-inflammatory cytokines, including adalimumab (for TNF-α neutralization) and tocilizumab (for IL-6 neutralization), have been shown to have some effectiveness for treating UC patients [15]. Using anti-IL-1β antibodies has also been shown to improve DSS-induced colitis in rodents [29]. In this current study, it was found that galangin had significant effects on decreasing three key pro-inflammatory cytokines (i.e., TNF-α, IL-1β, and IL-6) during colitis, which is supported by previous in vivo studies investigating galangin’s anti-inflammatory effects in LPS-injected mouse brains [30] and fructose-fed rat livers [31]. The colonic MPO is a hallmark for the neutrophil cell infiltration. One previous study suggested that galangin does not affect the NADPH oxidase activity, but it does strongly inhibit MPO activity in neutrophils [32]. The current study also found that the elevation in MPO activity induced by DSS was inhibited by galangin. Accompanying the histological analysis, this inferred that galangin was able to help prevent inflammatory cell infiltrations.

Autophagy is a physiological process that results in autophagosomal/lysosomal degradation of damaged cellular organelles or cytoplasmic contents [33]. Increasing evidence suggests that autophagy contributes to the homeostasis of the gastrointestinal system, as it is involved in intestinal cell development, tissue repair, and programmed cell death. The autophagy-related (Atg) genes control autophagosome formation through the Atg12-Atg5 and LC3-II (Atg8-II) complexes, and recent studies have noted that knockout of Atg genes in mice caused aggravation of colitis [34]. Therefore, activation of autophagy by dietary flavonoids/polyphenols could have great potential for controlling colitis. For example, one previous study demonstrated that a polyphenolic extract from mango (*Mangifera Indica* L.) had anti-colitis properties in a DSS-treated rat model, and this was meditated by its pro-autophagic activity [35]. Another study using a new synthetic flavonoid, GL-V9, showed it protected against colitis-associated colorectal cancer via activation of autophagy [36]. Galangin is a known natural inducer for autophagy, in many cell types, including hepatocellular carcinoma (HCC) [37], colon cancer [38], and oesophageal carcinoma cells [39]. Although few reports have noted the effects of galangin on cellular autophagy during colitis, this current study has shown for the first time that galangin can restore the impaired autophagy in DSS-treated mice, which is a promising therapeutic target for the treatment of UC. However, this study only focused on the effects of autophagy induction by galangin in the colon, and further studies investigating the interactions between autophagy and other cell signaling pathways, such as inflammatory regulation of cell apoptosis, should be performed in the future.

It has been shown that the pathogenesis of IBD involves both genetic and environmental factors, and studies have also highlighted the important role of the intestinal microbiota. Dietary supplements with natural products, including flavonoids, have been shown to have a certain potential in maintaining and/or reshaping the gut microbiome [40]. Despite the fact that most flavonoids have poor absorption, the gut microbiome is still largely affected by flavonoids because they have a long dwell time in the gut. The modulating effects on the gut microbiome have been widely recognized as the major contributors to the beneficial effects of flavonoids. In a prior study, galangin was found to have prebiotic properties, on the basis of its ability to enhance the production of anti-inflammatory substances produced by *Bifidobacterium adolescentis*—a commensal bacteria often isolated from the human large intestine [41]. In parallel, this current study noted that galangin treatment increased the gut bacterial diversity in colitic mice and increased the relative abundance of several beneficial bacterial strains, including *Mucispirillum* spp., *Bacteroides* spp., and *Anaerotruncus* spp. [42]. These results are in line with previous findings from other groups that assessed the gastrointestinal protective effects of bee propolis and galangal [43]. SCFAs are known energy sources for gut microbes, as well as for colonic epithelial cells, both of which play key important roles in maintaining gut hemostasis. Similar to previous studies, this current study demonstrated that intestinal SCFAs were decreased following DSS treatment, and galangin treatment was able to reverse these changes by increasing the acetate and butyrate levels. It was also noted that galangin enriched some specific bacteria populations that were able to promote the production of SCFAs, such as *Butyricimonas* spp., which inferred that the protective effect of galangin against DSS-induced colitis was mechanistically related to its effects of increasing SCFA production mediated by its remodeling effects on the gut microbiota.

We acknowledged that our study has also some limitations. First, although a DSS-induced UC model was applied in this study, the real cause of human IBD is still elusive and cannot be really mimicked. Second, we only observed the acute colitic symptoms in the mice, and this time period of observation might be not enough to assess the potential clinical outcomes by galangin intervention.

## 5. Conclusions

Taken together, the results of this study have shown that the administration of galangin to mice was able to alleviate acute UC-like symptoms. As a natural dietary bioflavonoid, galangin demonstrated good anti-inflammatory effects by inhibiting inflammatory cytokines as well as inflammatory cell infiltrations. It was also noted that galangin restored the impaired autophagy in these colitic mice. Moreover, it was demonstrated that galangin could increase the diversity of the gut microbiota diversity and promote SCFA production. As a result, galangin has demonstrated great potential for use in the prevention of UC.

## References

[1] Shouval D.S., Rufo P.A. (2017). The Role of Environmental Factors in the Pathogenesis of Inflammatory Bowel Diseases: A Review. JAMA Pediatr..

[2] Vindigni S.M., Zisman T.L., Suskind D.L., Damman C.J. (2016). The intestinal microbiome, barrier function, and immune system in inflammatory bowel disease: A tripartite pathophysiological circuit with implications for new therapeutic directions. Therap. Adv. Gastroenterol..

[3] M’Koma A.E., Moses H.L., Adunyah S.E. (2011). Inflammatory bowel disease-associated colorectal cancer: Proctocolectomy and mucosectomy do not necessarily eliminate pouch-related cancer incidences. Int. J. Colorectal Dis..

[4] Carding S., Verbeke K., Vipond D.T., Corfe B.M., Owen L.J. (2015). Dysbiosis of the gut microbiota in disease. Microb. Ecol. Health Dis..

[5] Lau W.L., Vaziri N.D. (2019). Gut microbial short-chain fatty acids and the risk of diabetes. Nat. Rev. Nephrol..

[6] Sunkara T., Rawla P., Ofosu A., Gaduputi V. (2018). Fecal microbiota transplant-a new frontier in inflammatory bowel disease. J. Inflamm. Res..

[7] Zmora N., Suez J., Elinav E. (2019). You are what you eat: Diet, health and the gut microbiota. Nat. Rev. Gastrol. Hepatol..

[8] Treml J., Smejkal K. (2016). Flavonoids as Potent Scavengers of Hydroxyl Radicals. Compr. Rev. Food Sci..

[9] Chirumbolo S. (2015). Anti-inflammatory property of propolis. J. Clin. Biochem. Nutr..

[10] Wang K., Jin X., Li Q., Sawaya A., Le Leu R.K., Conlon M.A., Wu L., Hu F. (2018). Propolis from Different Geographic Origins Decreases Intestinal Inflammation and Bacteroides spp. Populations in a Model of DSS-Induced Colitis. Mol. Nutr. Food Res..

[11] Wang K., Jin X., You M., Tian W., Le Leu R.K., Topping D.L., Conlon M.A., Wu L., Hu F. (2017). Dietary Propolis Ameliorates Dextran Sulfate Sodium-Induced Colitis and Modulates the Gut Microbiota in Rats Fed a Western Diet. Nutrients.

[12] Fang D., Xiong Z., Xu J., Yin J., Luo R. (2019). Chemopreventive mechanisms of galangin against hepatocellular carcinoma: A review. Biomed. Pharmacother..

[13] Sangaraju R., Nalban N., Alavala S., Rajendran V., Jerald M.K., Sistla R. (2019). Protective effect of galangin against dextran sulfate sodium (DSS)-induced ulcerative colitis in Balb/c mice. Inflamm. Res..

[14] Lassen K.G., McKenzie C.I., Mari M., Murano T., Begun J., Baxt L.A., Goel G., Villablanca E.J., Kuo S.Y., Huang H. (2016). Genetic Coding Variant in GPR65 Alters Lysosomal pH and Links Lysosomal Dysfunction with Colitis Risk. Immunity.

[15] Sahu B.D., Kumar J.M., Sistla R. (2016). Fisetin, a dietary flavonoid, ameliorates experimental colitis in mice: Relevance of NF-κB signaling. J. Nutr. Biochem..

[16] Hu L., Wu C., Zhang Z., Liu M., Maruthi Prasad E., Chen Y., Wang K. (2019). Pinocembrin protects against dextran sulfate sodium-induced rats colitis by ameliorating inflammation, improving barrier function and modulating gut microbiota. Front. Physiol..

[17] Russo A., Longo R., Vanella A. (2002). Antioxidant activity of propolis: Role of caffeic acid phenethyl ester and galangin. Fitoterapia.

[18] Kose L.P., Gulcin I., Goren A.C., Namiesnik J., Martinez-Ayala A.L., Gorinstein S. (2015). LC-MS/MS analysis, antioxidant and anticholinergic properties of galanga (Alpinia officinarum Hance) rhizomes. Ind. Crop Prod..

[19] (2019). 46th ESAO Congress 3-7 September 2019 Hannover, Germany Abstracts. Int. J. Artif. Organs..

[20] Rawla P., Sunkara T., Raj J.P. (2018). Role of biologics and biosimilars in inflammatory bowel disease: Current trends and future perspectives. J. Inflamm. Res..

[21] Tung J., Loftus E.V., Freese D.K., El-Youssef M., Zinsmeister A.R., Melton L.J., Harmsen W.S., Sandborn W.J., Faubion W.A. (2006). A population-based study of the frequency of corticosteroid resistance and dependence in pediatric patients with Crohn’s disease and ulcerative colitis. Inflamm. Bowel Dis..

[22] Kim J.J., Shajib M.S., Manocha M.M., Khan W.I. (2012). Investigating intestinal inflammation in DSS-induced model of IBD. J. Vis. Exp..

[23] Solomon L., Mansor S., Mallon P., Donnelly E., Hoper M., Loughrey M., Gardiner K. (2010). The dextran sulphate sodium (DSS) model of colitis: An overview. Comp. Clin. Pathol..

[24] Al-Rejaie S.S., Abuohashish H.M., Al-Enazi M.M., Al-Assaf A.H., Parmar M.Y., Ahmed M.M. (2013). Protective effect of naringenin on acetic acid-induced ulcerative colitis in rats. World J. Gastroenterol..

[25] Zhang Z.C., Li S., Cao H.Y., Shen P., Liu J.X., Fu Y.H., Cao Y.G., Zhang N.S. (2019). The protective role of phloretin against dextran sulfate sodium-induced ulcerative colitis in mice. Food Funct..

[26] Karrasch T., Kim J.S., Jang B.I., Jobin C. (2007). The flavonoid luteolin worsens chemical-induced colitis in NF-kappaB(EGFP) transgenic mice through blockade of NF-kappaB-dependent protective molecules. PLoS ONE.

[27] Azuma Y.T., Matsuo Y., Kuwamura M., Yancopoulos G.D., Valenzuela D.M., Murphy A.J., Nakajima H., Karow M., Takeuchi T. (2010). Interleukin-19 Protects Mice from Innate-mediated Colonic Inflammation. Inflamm. Bowel Dis..

[28] Kim T.W., Seo J.N., Suh Y.H., Park H.J., Kim J.H., Kim J.Y., Oh K.I. (2006). Involvement of lymphocytes in dextran sulfate sodium-induced experimental colitis. World J. Gastroenterol..

[29] Arai Y., Takanashi H., Kitagawa H., Okayasu I. (1998). Involvement of interleuking-1 in the development of ulceratie colitis inducted by dextran sulfate sodiium in mice. Cytokine.

[30] Choi M.J., Lee E.J., Park J.S., Kim S.N., Park E.M., Kim H.S. (2017). Anti-inflammatory mechanism of galangin in lipopolysaccharide-stimulated microglia: Critical role of PPAR-gamma signaling pathway. Biochem. Pharmacol..

[31] Sivakumar A.S., Anuradha C.V. (2011). Effect of galangin supplementation on oxidative damage and inflammatory changes in fructose-fed rat liver. Chem. Biol. Interact..

[32] Santos E.O., Kabeya L.M., Figueiredo-Rinhel A.S., Marchi L.F., Andrade M.F., Piatesi F., Paoliello-Paschoalato A.B., Azzolini A.E., Lucisano-Valim Y.M. (2014). Flavonols modulate the effector functions of healthy individuals’ immune complex-stimulated neutrophils: A therapeutic perspective for rheumatoid arthritis. Int. Immunopharmacol..

[33] Levine B., Yuan J. (2005). Autophagy in cell death: An innocent convict?. J. Clin. Investig..

[34] Grizotte-Lake M., Vaishnava S. (2018). Autophagy: Suicide Prevention Hotline for the Gut Epithelium. Cell Host Microbe.

[35] Kim H., Krenek K.A., Fang C., Minamoto Y., Markel M.E., Suchodolski J.S., Talcott S.T., Mertens-Talcott S.U. (2018). Polyphenolic derivatives from mango (Mangifera Indica L.) modulate fecal microbiome, short-chain fatty acids production and the HDAC1/AMPK/LC3 axis in rats with DSS-induced colitis. J. Funct. Foods.

[36] Zhao Y., Guo Q.L., Zhao K., Zhou Y.X., Li W.J., Pan C.Y., Qiang L., Li Z.Y., Lu N. (2018). Small molecule GL-V9 protects against colitis-associated colorectal cancer by limiting NLRP3 inflammasome through autophagy. Oncoimmunology.

[37] Li X., Wang Y.J., Xiong Y.Z., Wu J., Ding H., Chen X.Y., Lan L.B., Zhang H.T. (2016). Galangin Induces Autophagy via Deacetylation of LC3 by SIRT1 in HepG2 Cells. Sci. Rep..

[38] Ha T.K., Kim M.E., Yoon J.H., Bae S.J., Yeom J., Lee J.S. (2013). Galangin induces human colon cancer cell death via the mitochondrial dysfunction and caspase-dependent pathway. Exp. Biol. Med..

[39] Ren K.W., Zhang W.Z., Wu G., Ren J.Z., Lu H.B., Li Z.M., Han X.W. (2016). Synergistic anti-cancer effects of galangin and berberine through apoptosis induction and proliferation inhibition in oesophageal carcinoma cells. Biomed. Pharmacother..

[40] Joo M., Kim H.S., Kwon T.H., Palikhe A., Zaw T.S., Jeong J.H., Sohn U.D. (2015). Anti-inflammatory Effects of Flavonoids on TNBS-induced Colitis of Rats. Korean. J. Physiol. Pharmacol..

[41] Kawabata K., Sugiyama Y., Sakano T., Ohigashi H. (2013). Flavonols enhanced production of anti-inflammatory substance(s) by Bifidobacterium adolescentis: Prebiotic actions of galangin, quercetin, and fisetin. Biofactors.

[42] Liu G., Yan W.X., Ding S.J., Jiang H.M., Ma Y., Wang H., Fang J. (2018). Effects of IRW and IQW on Oxidative Stress and Gut Microbiota in Dextran Sodium Sulfate-Induced Colitis. Cell Physiol. Biochem..

[43] da Silva L.M., de Souza P., Al Jaouni S.K., Harakeh S., Golbabapour S., de Andrade S.F. (2018). Propolis and Its Potential to Treat Gastrointestinal Disorders. Evid.-Based Compl. Alt..

